# Adipokines in oral squamous cell carcinoma—a narrative overview

**DOI:** 10.1038/s41405-026-00444-x

**Published:** 2026-05-14

**Authors:** Pavithra Velusamy, Mary Mathew, Adarsh Kudva, Chetana Chandrashekar, Srikanth Gadicherla, Yoithaprabhunath Thuckanaickenpalayam Ragunathan, Sri Chinthu Kenniyan Kumar, Monica Charlotte Solomon

**Affiliations:** 1 Department of Oral and Maxillofacial Pathology and Oral Microbiology, Manipal College of Dental Sciences, Manipal Academy of Higher Education, Manipal, India; 2 Department of Pathology, Kasturba Medical College, Manipal Academy of Higher Education, Manipal, India; 3 Department of Oral and Maxillofacial Surgery, Manipal College Of Dental Sciences, Manipal Academy of Higher Education, Manipal, India; 4 Department Of Oral and Maxillofacial Pathology and Oral Microbiology, Vivekanandha Dental College for Women, Namakkal, Tamil Nadu India; 5 Department of Oral and Maxillofacial Pathology and Oral Microbiology, KSR Institute of Dental Science and Research, Tiruchengode, Tamil Nadu India

**Keywords:** Oral cancer detection, Oral cancer detection

## Abstract

**Objectives:**

To identify and summarize the types of adipokines associated with Oral Squamous Cell Carcinoma (OSCC) pathogenesis and prognosis.

**Materials and Methods:**

A comprehensive literature search was done involving Scopus, PubMed, and Embase. Studies involving adipokines in OSCC conducted in humans were included.

**Results:**

Adipokines have diverse and stage-dependent effects on OSCC, including regulation of inflammation, angiogenesis, hypoxia, and tumour progression. Adipokines such as chemerin, resistin, leptin, and apelin contribute to tumour progression. Zinc alpha-2-glycoprotein and adiponectin were found to be antitumourigenic in OSCC. Methodological heterogeneity and a lack of standardized outcome measures limit comparability across studies to establish the definitive role of adipokines in OSCC.

**Conclusion:**

Adipokines shape OSCC biology through diverse, stage-specific mechanisms, making them potential biomarkers of disease progression. The adipokines reported in OSCC require validation through long-term prospective studies and addressing methodological heterogeneity to serve as prognostic markers.

## Introduction

Oral squamous cell carcinoma (OSCC) is the most commonly diagnosed oral cancer and is associated with a significant health burden, with a marked geographic and demographic distribution. Oral cancer is ranked the fourth most common cancer in the Southeast Asian population, with 149,102 new cases diagnosed in 2018. OSCC is frequently diagnosed in developing South-Asian countries such as Sri Lanka, Pakistan, India, and Bangladesh. This marked prevalence among specific populations is attributed to the use of tobacco, betel quid, and alcohol, which are significant risk factors for OSCC [1].

Despite multiple advances in diagnostic and therapeutic approaches, the survival rate remains suboptimal. The molecular mechanisms underlying OSCC pathogenesis must be better understood to facilitate early diagnosis and improve prognosis. The interlink between immunity, inflammation, and cancer has been spotlighted recently. Transcription factors regulate the expression of inflammatory mediators and play crucial roles in the survival and proliferation of cancer cells. Cancer cells induce an inflammatory state in the tumour stroma, promoting cancer cell growth, invasion, and metastasis [2].

Due to chronic inflammation, inflammatory mediators such as cytokines, prostaglandins, and reactive oxygen species(ROS) accumulate in the tumour microenvironment [3]. These mediators generate reciprocating interactions between tumour cells and their stroma, promoting cancer cell proliferation and tumour progression [4]. Adipokines are inflammatory mediators that adipose tissue produces and play essential roles in metabolism, immunity, and inflammation. Adipokines influence the tumour microenvironment, undergo metabolic reprogramming, and drive epithelial-mesenchymal transition(EMT), promoting tumour cell proliferation, invasion, and metastasis [5–7]. This review summarizes the adipokines studied in OSCC with respect to pathogenesis and prognosis.

## Methods

**Fig. 1:**
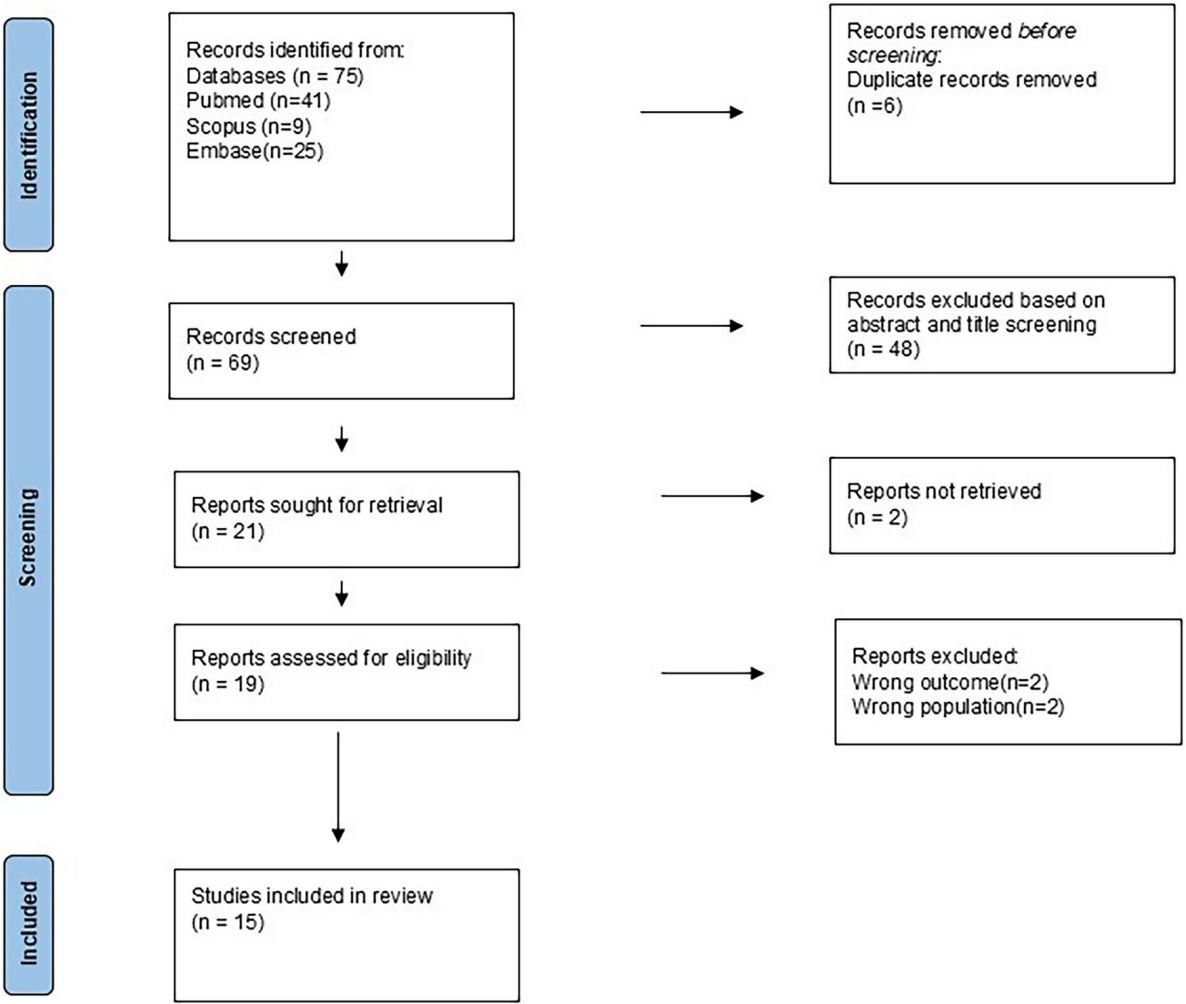
Study selection process. The figure shows the detailed study selection process used to identify articles on the role of adipokines in OSCC.

A literature search of published articles was performed across PubMed, Scopus, and Embase till February 2025. The literature search was conducted using the combination of keywords “Adipokines” OR “Adipocytokine” AND “Oral Squamous Cell Carcinoma”. In addition, articles were handpicked. Studies that analysed the role of adipokines in OSCC in humans were included. Studies on adipokines in other cancers, narrative reviews, systematic reviews, editorials, grey literature, and studies in other languages were excluded. Initially, 75 articles were retrieved through the electronic database search. Of 75 articles, 6 duplicates were removed, leaving 69 articles for abstract and title screening. Based on abstract and title screening, 48 articles were excluded. Further, 21 articles were sought for retrieval, of which 2 articles lacked full text. A total of 19 articles were assessed for eligibility by full-text reading. After final screening by full-text reading, 15 articles were selected (Fig. 1). Due to high heterogeneity in the methodology of the included studies, a systematic review was not feasible. Henceforth, we presented the results of the search as a narrative overview, and adherence to PRISMA reporting guidelines was not applicable.

## Results and discussion

Oral squamous cell carcinoma develops through a multistage process driven by exposure to carcinogens, genetic mutations, and chronic inflammation. Prolonged exposure to carcinogens induces DNA damage, oxidative stress, and the activation of signalling pathways, creating a microenvironment favourable for malignant transformation. Tumour progression is driven by the interplay among malignant cells, immune cells, stromal fibroblasts, the extracellular matrix, and metabolic mediators. Chronic inflammation is now recognized as the key mechanism driving cellular proliferation, angiogenesis, immune evasion, and metastasis. Comprehending this process is essential for understanding the pathophysiology of cancer and developing therapeutic targets [8].

Adipose tissue comprises adipocytes, endothelial cells, fibroblasts, mast cells, immune cells, and stem cells. They were considered passive energy reservoirs, but are now regarded as endocrine organ that play important roles in cancer through the secretion of adipokines [9]. Adipokines play various roles in cancer, including immune responses, the promotion of inflammation, enhanced metabolic activity, and angiogenesis. The inflammatory milieu provided by adipokines facilitates reciprocal signalling between tumour cells and their surrounding stroma, creating a microenvironment conducive to tumour progression [10].

**Fig. 2:**
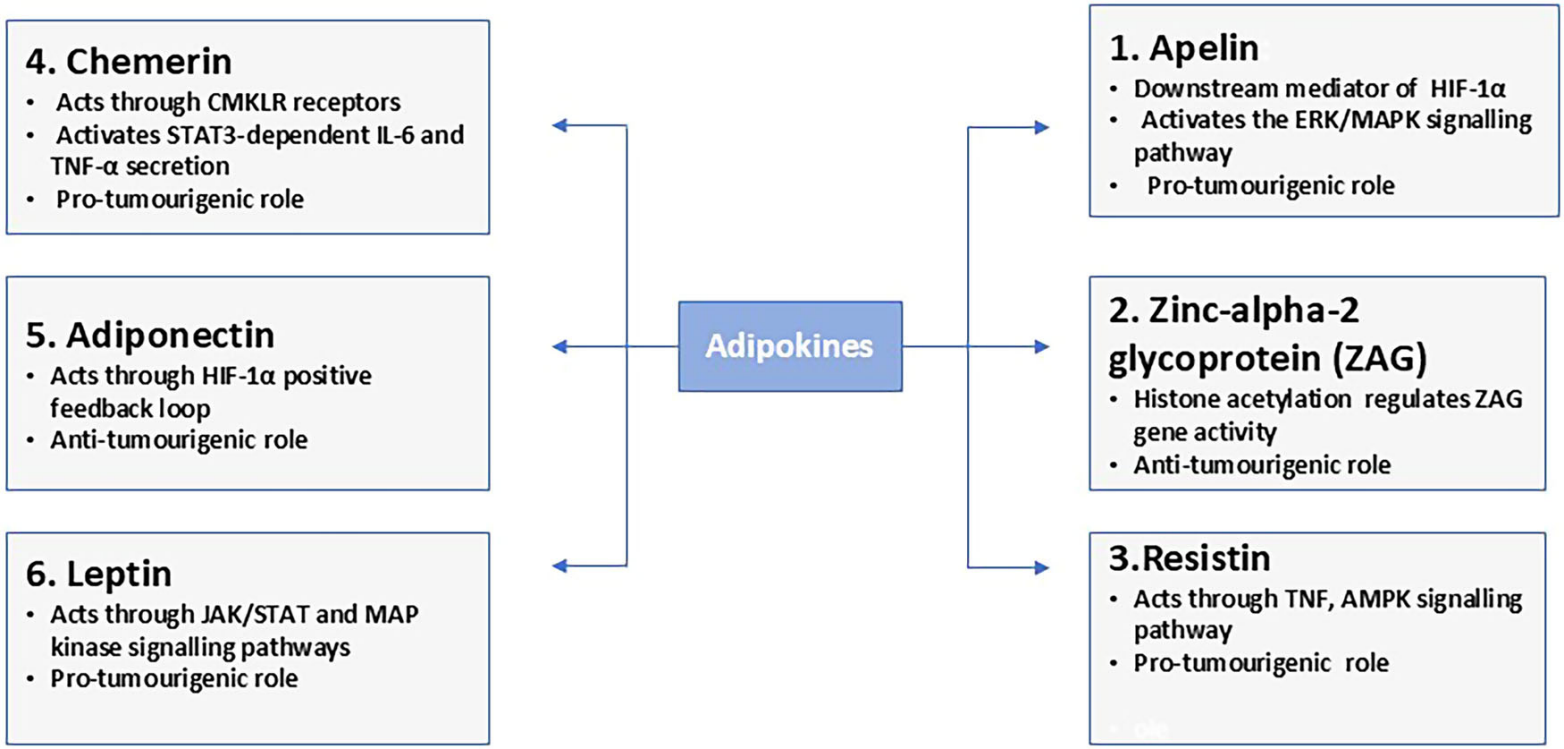
Functional role of adipokines in OSCC. The figure outlines the functional roles of various adipokines along with their key signalling pathways in OSCC.

When tumour cells start proliferating rapidly, they need a surplus of energy for rapid cell division and DNA replication. Tumour cells interact with adipose tissue, altering adipocyte function and paracrine signalling. Tumour cells that co-opt adipocytes to meet their metabolic needs are called cancer-associated adipocytes (CAA). Signalling between adipocytes and cancer cells stimulates lipolysis in adipose tissue which subsequently releases fatty acids. These fatty acids enter cancer cells and bind to the fatty acid receptor binding site. Cancer cells rely more on fatty acids for energy, which results in greater ATP production, as glucose alone cannot meet the needs of the growing mass. Fatty acid oxidation is required to meet the tumour’s energy needs during growth, extracellular matrix (ECM) remodelling, and metastasis [11]. Hypoxia triggers increased lipid uptake through HIF-1α by inducing FABP3/4 expression along with the expression of adipophilin, a lipid structural protein. Fatty acids, in addition to their role as energy reservoir, are also involved in membrane lipid synthesis in cancer cells, which affects cellular signalling [12]. Inhibition of lipolysis, lowering free fatty acid levels, has been shown to reduce cancer pathogenicity, suggesting therapeutic implications for the use of anti-lipolytics in cancer treatment [13]. In this review, we identified six key adipokines apelin, chemerin, resistin (RETN), leptin, adiponectin, and zinc-alpha-2 glycoprotein (ZAG) that were studied in the context of OSCC and are discussed below (Fig. 2).

### Apelin

Apelin is a bioactive peptide involved in angiogenesis, cell proliferation, and migration in various types of cancer. Apelin expression is induced under hypoxic conditions, and the Apelin gene is a downstream mediator of HIF-1α in OSCC. Apelin activates the ERK/MAPK signalling pathway, suggesting its role in tumour growth. High expression of Apelin was significantly associated with tumour recurrence and poor disease-free survival. Apelin was an independent prognostic factor for disease-free survival, along with age and lymph node metastasis. Apelin promoted the proliferation and migration of OSCC cells in a dose-dependent manner [14]. These findings underscore the importance of apelin in promoting tumour growth and as a prognostic factor in OSCC, warranting further studies to identify its role in tumour progression.

### Zinc-alpha-2 glycoprotein (ZAG)

Zinc-alpha-2 glycoprotein (ZAG) is an adipokine that is actively involved in the early stages of OSCC. The exact mechanism regulating ZAG is unclear. However, its expression is increased by histone acetylation, which alters chromatin structure and regulates ZAG gene activity. On the other hand, histone deacetylation is associated with reduced ZAG expression in pancreatic cancer [15]. High ZAG expression in the early stages of oropharyngeal squamous cell carcinoma was associated with long recurrence-free survival [16]. ZAG promotes tumour proliferation and mucosal breakdown by inducing an immune response against tumour antigens. ZAG downregulation is linked to metastasis and poor prognosis in head and neck carcinomas [17]. ZAG expression was seen in early-stage OSCC cases, whereas none of the advanced-stage cases showed expression. ZAG was significantly associated with small tumour size, absence of lymph node involvement, early clinical stage, and less differentiated tumours [18]. ZAG can be a useful biomarker for predicting early-stage cancers, as its expression is influenced by epigenetic mechanisms, such as histone modifications, which play a vital role in tumour behaviour. Thus, ZAG’s cytoplasmic expression and association with early-stage and loss of expression in advanced OSCC cases can describe its role in limiting the tumour’s spread.

### Resistin(RETN)

Resistin (RETN) is an adipokine that has been studied in the context of obesity and inflammation. RETN gene polymorphisms have been identified in colon, breast, and lung cancer [19–21]. Bioinformatic analysis of the RETN gene revealed an interaction with TNF, which is linked to non-alcoholic fatty liver disease (NAFLD) and the AMPK signalling pathway, which connects inflammation to cancer. On homologous modelling of the RETN gene, mutations in *RETN-*specific G-A were associated with melanoma, glioblastoma multiforme, and pancreatic cancer. The RETN gene interacts significantly with multiple pathways involved in carcinogenesis. Pathway enrichment analysis revealed that RETN regulates adipogenesis, FOXO-mediated transcription, oxidative stress, gene expression, and RNA polymerase II transcription. These pathways play crucial roles in regulating cell growth, modulating stress responses, and inflammation, all of which are key events in carcinogenesis [22].

Yang WH et al. [23] found that carriers of the RETN gene polymorphism who consumed betel nuts had a higher risk of developing OSCC than those without any habit. Compared with G/G homozygotes, OSCC patients with the A/A homozygote of the *RETN* rs3219175 polymorphism had a high risk of having advanced tumours. There was no difference in RETN gene variants between cancer patients and controls [23]. Arif K et al studied the distribution of the RETN rs3219175 single-nucleotide polymorphism among OSCC patients. The GG genotype was the most common, followed by the GA genotype, while the AA genotype was absent in both OSCC cases and controls. A significant association was found between the GG and GA genotypes and oral cancer risk [22]. The RETN gene may not be solely responsible for cancer, but also requires some triggers, such as smoking and alcohol consumption, to activate the gene [23]. These studies highlight the importance of RETN gene screening and environmental risk assessment in elucidating its role as a diagnostic marker for OSCC. RETN has been studied primarily through polymorphism analysis rather than protein analysis, as with other adipokines. Although RETN plays a protumourigenic role through inflammatory and metabolic signalling networks, its applicability as a standalone marker is constrained by a lack of protein-level validation.

### Chemerin

Chemerin, a multifunctional adipokine, regulates angiogenesis, cell proliferation, inflammation, and lipid metabolism. Chemerin mediates angiogenesis, an important hallmark feature in cancer, by acting through CMKLR receptors on endothelial cells in a paracrine manner [8]. Chemerin contributed to the proinflammatory milieu in the tumour microenvironment by activating proinflammatory cytokines and recruiting immune cells that promote tumour progression. A study by Lu Z et al. [24] showed that serum chemerin levels were elevated in patients with lymph node involvement compared with those without lymph node involvement in OSCC. Serum chemerin levels showed significant correlations with IL-6, GM-CSF, TNF-α, and VEGF levels in OSCC patients. Treatment with exogenous recombinant chemerin increased IL-6 and TNF-α secretion by activating STAT3 in OSCC cell lines. Chemerin enhanced the migration and invasion of OSCC cells, which were reduced upon neutralizing antibody blockade of IL-6 and TNF-α [24]. Similarly, in Ghallab NA 2017 study the serum and salivary levels of chemerin and MMP-9 were significantly elevated in OSCC compared with the Oral Premalignant Lesion (OPML) group and healthy controls [25].

Chemerin showed strong immunohistochemical expression in tumoural tissues, particularly in the cytoplasm, whereas adjacent normal tissues showed weak or absent expression. High chemerin expression correlated with poor differentiation, lymph node involvement, and advanced clinical stage. Chemerin significantly correlated with microvessel density(MVD), indicating that chemerin can mediate angiogenesis. Patients having moderate to strong expression of chemerin had a poor cancer-related survival on multivariate analysis [8]. Compared with other adipokines, Chemerin shows a consistent protumourigenic profile in OSCC, supported by serum, saliva, and tissue studies, as well as in vitro studies, that have correlated with angiogenesis, lymph node metastasis, and poor survival.

### Adiponectin

Adiponectin, an adipokine that predominantly acts through its receptors AdipoR1 and AdipoR2 has antidiabetic and anti-inflammatory effects. Guo et al. [26] reported low serum adiponectin levels in tongue squamous cell carcinoma (TSCC) patients than in controls. Low adiponectin levels were inversely associated with advanced histological grade and lymph node metastasis. Adiponectin levels in tumour tissues decreased with advancing stage, whereas adiponectin receptor expression remained unchanged. Furthermore, adiponectin inhibited the migration of SCC15 cells in vitro [26]. Guo et al.‘s 2023 study expanded their observations from their early study conducted in 2013, offering more profound insights into the molecular mechanism of adiponectin in TSCC. The 2023 study revealed that Adiponectin and its receptor AdipoR1 have been upregulated in early-stage TSCC and under hypoxic conditions. A paradoxical decrease in adiponectin mRNA levels was seen with increased protein levels, suggesting post-transcriptional regulation. Lentiviral knockdown of HIF-1α decreased the levels of adiponectin and AdipoR. Treatment with recombinant globular adiponectin upregulated HIF-1α, suggesting a positive feedback loop [27]. Guo et al. in 2013 showed that low serum and tissue adiponectin levels correlated with advanced tumour stage and inhibited the proliferation of TSCC cells in vitro. Whereas the 2023 study showed a loss of adiponectin mRNA levels despite increased protein expression in early stages, suggesting a protective mechanism that preserves adiponectin. In later stages, adiponectin protein levels decreased due to hypoxia and metabolic stress in the tumour microenvironment. Overall, adiponectin can exert antitumourigenic effects by being expressed in the early stages of TSCC, but its effect diminishes in advanced stages due to hypoxia-driven resistance mechanisms.

### Leptin

Leptin contributes to tumourigenesis by playing a pivotal role in inflammation and angiogenesis. Leptin binds to its receptor on keratinocytes, inducing cell proliferation by activating the JAK/STAT and MAP kinase signalling pathways [28–30]. Leptin appears to mediate the HIF-1α pathway and is more active in the early stage of carcinogenesis [31]. Activation of these pathways induces cell proliferation, angiogenesis, and the inflammatory response. In addition, leptin modulates the tumour microenvironment by activating endothelial cells and recruiting macrophages and monocytes, thereby promoting angiogenesis and the release of proinflammatory cytokines [32, 33]. Leptin and leptin receptor polymorphisms contribute to the genetic risk factors for the development of OSCC. Leptin induced cell proliferation and migration and inhibited apoptosis in vitro. Leptin-treated OSCC cells exhibited increased expression of E-cadherin, Col1A1, MMP-2, MMP-9, and miR-210, which facilitated tumour progression. Animal models exhibit increased serum leptin levels and higher expression of leptin and its receptor in tissues. The expression of hypoxia-inducible factor 1-alpha messenger RNA, leptin, and its receptor was increased in human OSCC tissues [31].

In Yapijakis C et al. 2008 study, Leptin (LEP 2548 G/A) polymorphism was significantly increased in patients with advanced-stage OSCC and patients with a family history of cancer, and in patients without tobacco and alcohol consumption. The LEPR Q223R G/G genotype was associated with an increased risk of OSCC. Further, they stated that leptin has a tumour-promoting role and can induce angiogenesis and metastasis [34]. Hussain S et al. [35] reported that the leptin gene (G2548A) polymorphism, involving the homozygous mutant A allele, and the leptin receptor (LEPR A668G) polymorphism, involving the homozygous mutant G allele, were significantly increased in OSCC compared to controls in a cohort of the Indian subpopulation [35]. However, in the Hung WC et al. [36] study, the single-nucleotide polymorphisms in Leptin (LEP − 2548) and its receptor, LEPR K109R and LEPR Q223R, were not associated with increased risk of OSCC. Non–smokers with LEP-2548 G/A polymorphism had a statistically significant increased risk of developing OSCC compared to individuals with tobacco consumption [36].

Salivary and serum leptin levels were reduced in OSCC involving the buccal mucosa compared with controls. Weight loss was observed among cancer patients and correlated significantly with both histopathological grading and clinical staging. Cytokine-induced cachexia can produce negative leptin feedback to the hypothalamus, contributing to weight loss [37]. In Gharote HP et al.‘s [38] study, serum leptin levels correlated with tumour differentiation and BMI, with no significance for tumour stage [38]. Whereas in Sobrinho Santos et al’s [31] study, serum leptin levels in humans did not show a significant association and were only increased in the initial stages of OSCC [31].

Leptin is the most commonly studied adipokine in oral cancer, with respect to pathogenesis and prognosis. Animal studies showed that leptin induces cell proliferation, which was further confirmed in human OSCC tissues. Whereas clinical studies measuring serum and salivary leptin levels showed increased expression in the early stages, and its effect diminished later. Cancer-induced cachexia and systemic inflammation are known to suppress circulating leptin levels in advanced OSCC cases. Circulating leptin levels reflect overall metabolic status, whereas tissue expression reflects tumour-specific activity and may contribute to the heterogeneity of the results. Leptin and leptin receptor expression were increased in OSCC in some genetic polymorphism studies, but findings have been inconsistent across studies, with leptin-related polymorphisms acting as modifiers of risk rather than independent determinants.

Leptin plays a complex, stage-dependent role, and genetic susceptibility increases the risk of OSCC. Leptin levels vary across disease stages and are reduced in cachexia [37], elevated in initial stages [31], and influenced by the genotype expression [34]. Its involvement in tumour biology, genetic susceptibility, and interaction with environmental carcinogens makes leptin a proactive molecule in carcinogenesis. Leptin plays a predominant protumourigenic role, and a decline in the advanced stage may reflect the systemic metabolic changes rather than a tumour suppressive role.

Higher expression levels of Apelin [14] and Chemerin [8, 24, 25] correlated with poor prognosis in OSCC, suggesting a protumourogenic role. Patients with Resistin gene polymorphisms had an increased risk of developing OSCC, suggesting that Resistin is protumourogenic [22]. Zinc Alpha2 glycoprotein and adiponectin were seen only in the early stages of OSCC, and their effects diminished in the advanced stage, exerting an antitumourogenic role [18, 27]. Although leptin levels decline in later stages of OSCC, the overall evidence suggests that it predominantly plays a protumourogenic role in tumour initiation and progression [37, 38]. Overall, adipokines play a complex role in the pathogenesis of OSCC (Table 1).

**Table 1:** Lists the adipokines studied in OSCC.

Author, Country	Sample size	Adipokine studied	Methodology used	Outcome
Heo K et al. [14] Korea	OSCC - 62	Apelin	Tissue samples: Immunohistochemistry Western blot Cell lines: Cell culture and transfection, cell proliferation assay, invitro wound healing, transwell migration assay, RT-PCR	Stronger expression of apelin significantly correlated with tumour recurrence and disease-free survival. Under hypoxic conditions, apelin expression was upregulated, and exogenous apelin enhanced the proliferation and migration of OSCC cells.
Ali M et al. [18] Pakistan	OSCC- 120	Zinc- alpha 2 glycoprotein (ZAG)	Tissue samples: Immunohistochemistry	Early-stage OSCC cases(71%) were positive for immunohistochemistry, whereas cases in advanced stages had negative expression. High ZAG showed a significant association with smaller tumour size, lymph node involvement, early stages of OSCC, and less differentiated OSCC.
Yang W et al. [23] Taiwan	OSCC -935 Healthy controls - 1200	Resistin (RETN)	Blood samples: Genotyping Assay	The rs3219175 polymorphism of A/A homozygosity in OSCC had a high risk for advanced tumour size (>T2). A/T/G/G haplotype increased the risk of OSCC by 1.376 times.
Arif K et al. [22] Pakistan	OSCC- 35 Healthy controls – 35	Resistin (RETN)	Blood samples: a.RT- PCR b.HRM – High resolution melting curve analysis	Resistin rs3219175 polymorphism GG genotype was more frequent among OSCC (68%) than controls (54%) compared to GA genotype.
Wang N et al. [8] China	SCCOT -147 archived tissue samples,19 fresh tumoral tissues from SCCOT	Chemerin	Archived tissue samples: Immunohistochemistry Fresh tumoral tissues: Real-time quantitative transcription polymerase chain reaction (qRT-PCR)	Chemerin was overexpressed in SCCOT compared to non-cancerous tissue. Overexpression of Chemerin positively correlated with Microvessel Density(MVD). Patients with overexpression of Chemerin had shorter survival compared to those with low expression.
Ghallab N et al. [25] Egypt	15 – early stage OSCC 15 -OPML 15- Controls	Chemerin	Serum and saliva samples: ELISA	Serum and salivary levels of Chemerin and MMP-9 were significantly higher in the OSCC group compared to OPML and the control group.
LuZ et al. [24] China	OSCC LN+ve – 20 OSCC LN-ve – 20	Chemerin	Serum samples: a.ELISA: Chemerin b.Luminex liquid suspension assay: cytokines	Serum chemerin levels correlated with the levels of IL-6, GM-CSF, TNF-α, VEGF in OSCC patients. OSCC patients with lymph node metastasis had higher levels of cytokines compared to OSCC patients without lymph node metastasis.
Guo XH et al. [26] China	Serum: TSCC- 59 Controls- 50 Tissue: 37 – IHC 30 – Western Blot	Adiponectin	Serum samples: a.ELISA Tissue samples: b.Immunohistochemistry c. Western blot SCC15 cell line: d.RT-PCR e.Cell culture and proliferation assay f.Migration assay	Serum adiponectin levels were lower in TSCC compared to controls. Adiponectin in tumoral tissue decreased as the TNM stage increased. Adiponectin inhibited the migration of the SCC15 cell line in vitro.
Guo XH et al. [27] China	TSCC-46	Adiponectin	Tissue samples: a. RT-PCR b.Western blot c.Immunohistochemistry Cell lines (SCC9, SCC 15): c.Gene manipulation d.Cell proliferation assay e.Wound healing assay.	Protein expression of Adiponectin and AdipoR1 was upregulated in the early stage of TSCC. Knocking down HIF-1α decreased the levels of Adiponectin and AdipoR1. Blockade of HIF-1α with recombinant globular adiponectin exhibits a synergistic anti-tumour effect
Gharote H et al. [38] India	OSCC- 31 Controls-28	Leptin	Serum samples: ELISA	Mean serum leptin levels were higher in the control group compared to the OSCC group.
Kaur J et al. [37] Belgium	OSCC-41 Control-40	Leptin	Serum Leptin: ELISA Salivary Leptin: Radioimmunoassay	Salivary and serum leptin levels were reduced in OSCC patients compared to controls.
Santos E et al. [31] Brazil	Tissue: OSCC- 26 Control -25 Serum: 11- OSCC 13 - Control	Leptin	Tissue: Immunohistochemistry of leptin & CD31 qRT-PCR Serum samples: ELISA Cell lines: Cell culture (SCC-9 & SCC-4) & treatment Cell proliferation & visibility Migration & invasion assay qRT-PCR	Higher rates of cell proliferation and migration, and reduced apoptosis were seen with leptin treatment. Protein expression of leptin and leptin receptor was significantly higher in OSCC cases. Serum leptin levels were increased in the initial stages of the disease. OSCC-induced mice had increased serum leptin levels in the dysplasia group.
Hussain S et al. [35] India	OSCC-306 Controls - 228	Leptin	Blood samples: a.Polymerase Chain Reaction and restriction fragment length polymorphism (PCR-RFLP) b.Genotyping of the leptin G2548A and leptin A668G (Q223R)	The Leptin gene G2548A homozygous mutant AA polymorphism and A668G homozygous mutant GG polymorphism were significantly increased in the OSCC patients compared to controls.
Hung W et al. [36] Taiwan	0SCC – 567, Control – 560	Leptin and Leptin receptor (LEPR)	Blood samples: Real-time PCR	LEP- 2548 G/A single-nucleotide polymorphisms were frequently seen in non–tobacco users compared to the tobacco users. Single Nucleotide Polymorphisms of LEP − 2548 G/A(rs7799039), LEPRK109R(rs1137100), and LEPRQ223R (rs1137101) were not associated with increased risk of oral cancer.
Yapijakis C et al. [34] Germany& Greece	OSCC – 150 Controls - 152	Leptin	Blood samples: Polymerase chain reaction-based restriction analysis	Homozygous high gene expression genotype A/A of the LEP 2548 G/A polymorphism was significantly increased in the patients with advanced OSCC stage, whereas low leptin binding genotype G/G of the LEPR Q223R polymorphism was increased in all stages of OSCC.

*OSCC* oral squamous cell carcinoma, *SCCOT* squamous cell carcinoma of the tongue, *PCR* polymerase chain reaction, *ELISA* enzyme-linked immunosorbent assay, *RT-PCR* reverse transcription polymerase chain reaction, *TSCC* tongue squamous cell carcinoma.

Absence of survival data, lack of justification for sample size, and the limited follow-up duration were some of the lacunae identified in these studies. Furthermore, exclusion criteria and confounders were not stated in some studies. The studies exhibit substantial methodological heterogeneity, making it challenging to interpret the complex role of adipokines in OSCC pathogenesis. Standardized methodologies enhance comparability across the studies. Adipokines are elevated in Obesity, Diabetes Mellitus, Metabolic Syndrome, Polycystic Ovarian Syndrome, and in certain types of cancer. Accounting for inflammatory oral conditions is crucial because these adipokines are already elevated in people with periodontitis. A comprehensive evaluation of these biomarkers, while controlling confounders, is essential to strengthen the role of adipokines in OSCC. Well-designed studies with larger sample sizes and long-term follow-up are necessary to validate these findings.

## Conclusion

This review highlights the importance of adipokines in the prognosis and pathogenesis of OSCC. Adipokines such as Apelin, Resistin, Leptin, and Chemerin played a protumourigenic role in OSCC. Some adipokines, such as Zinc Alpha2 glycoprotein and Adiponectin, have antitumourogenic potential. Longitudinal studies with standardized methodologies are essential to validate the complex role of adipokines in OSCC.

## Data Availability

All the data associated with this review are presented in the manuscript.

## References

[1] Sarode G, Maniyar N, Sarode SC, Jafer M, Patil S, Awan KH. Epidemiologic aspects of oral cancer. Dis Mon. 2020;66:100988. 10.1016/j.disamonth.2020.100988. 32605720 10.1016/j.disamonth.2020.100988

[2] Feller L, Altini M, Lemmer J. Inflammation in the context of oral cancer. Oral Oncol. 2013;49:887–92. 10.1016/j.oraloncology.2013.07.003. 23910564 10.1016/j.oraloncology.2013.07.003

[3] Colotta F, Allavena P, Sica A, Garlanda C, Mantovani A. Cancer-related inflammation, the seventh hallmark of cancer: links to genetic instability. Carcinogenesis. 2009;30:1073–81. 10.1093/carcin/bgp127. 19468060 10.1093/carcin/bgp127

[4] Hanahan D, Weinberg RA. Hallmarks of cancer: the next generation. Cell. 2011;144:646–74. 10.1016/j.cell.2011.02.013. 21376230 10.1016/j.cell.2011.02.013

[5] Wang X, Wang J, Zhao X, Zhang J, Zhang Y. The adipokines in oral cancer pathogenesis and their potential as a new therapeutic approach. Naunyn Schmiedebergs Arch Pharm. 2025;398:9623–39. 10.1007/s00210-025-03939-w. 10.1007/s00210-025-03939-w 40056203

[6] Kim JW, Kim JH, Lee YJ. The role of adipokines in tumor progression and its association with obesity. Biomedicines. 2024;12:97. 10.3390/biomedicines12010097. 10.3390/biomedicines12010097 PMC10813163 38255203

[7] Kounatidis D, Vallianou NG, Karampela I, Grivakou E, Dalamaga M. The intricate role of adipokines in cancer-related signaling and the tumor microenvironment: Insights for future research. Semin Cancer Biol. 2025;113:130–150. 10.1016/j.semcancer.2025.05.013. 10.1016/j.semcancer.2025.05.013 40412490

[8] Wang N, Wang QJ, Feng YY, Shang W, Cai M. Overexpression of chemerin was associated with tumour angiogenesis and poor clinical outcome in squamous cell carcinoma of the oral tongue. Clin Oral Investig. 2014;18:997–1004. 10.1007/s00784-013-1046-8. 23868294 10.1007/s00784-013-1046-8

[9] Jasinski-Bergner S, Kielstein H. Adipokines regulate the expression of tumour-relevant MicroRNAs. Obes Facts. 2019;12:211–25. 30999294 10.1159/000496625 PMC6547259

[10] Blüher M. Adipose tissue dysfunction in obesity. Exp Clin Endocrinol Diab. 2009;117:241–50. 10.1055/s-0029-1192044 19358089

[11] Mukherjee A, Bilecz AJ, Lengyel E. The adipocyte microenvironment and cancer. Cancer Metastasis Rev. 2022;41:575–87. 10.1007/s10555-022-10059-x. 35941408 10.1007/s10555-022-10059-x

[12] Lengyel E, Makowski L, DiGiovanni J, Kolonin MG. Cancer as a matter of fat. Crosstalk Adipose Tissue Tumours Trends Cancer. 2018;4:374–84. 10.1016/j.trecan.2018.03.004. 29709261 10.1016/j.trecan.2018.03.004 PMC5932630

[13] Currie E, Schulze A, Zechner R, Walther TC, Farese RV Jr. Cellular fatty acid metabolism and cancer. Cell Metab. 2013;18:153–61. 10.1016/j.cmet.2013.05.017. 23791484 10.1016/j.cmet.2013.05.017 PMC3742569

[14] Heo K, Kim YH, Sung HJ, Li HY, Yoo CW, Kim JY, et al. Hypoxia-induced up-regulation of apelin is associated with a poor prognosis in oral squamous cell carcinoma patients. Oral Oncol. 2012;48:500–6. 10.1016/j.oraloncology.2011.12.015. 22285858 10.1016/j.oraloncology.2011.12.015

[15] Tang H, Wu Y, Qin Y, Wang H, Wang L, Guan X, et al. Reduction of AZGP1 predicts poor prognosis in esophageal squamous cell carcinoma patients in Northern China. Onco-Targets Ther. 2016;10:85–94. 10.2147/OTT.S1139323. 28053542 10.2147/OTT.S113932 PMC5189973

[16] Poropatich K, Paunesku T, Zander A, Wray B, Schipma M, Dalal P, et al. Elemental Zn and its binding protein zinc-α2-glycoprotein are elevated in HPV-positive oropharyngeal squamous cell carcinoma. Sci Rep. 2019;9:16965. 10.1038/s41598-019-53268-1. 31740720 10.1038/s41598-019-53268-1 PMC6861298

[17] Vidotto A, Henrique T, Raposo LS, Maniglia JV, Tajara EH. Salivary and serum proteomics in head and neck carcinomas: before and after surgery and radiotherapy. Cancer Biomark. 2010;8:95–107. 10.3233/cbm-2011-0205. 21896997 10.3233/CBM-2011-0205 PMC13015853

[18] Ali MF, Hosein M, Butt S, Siddiqui R. Zinc-alpha 2 glycoprotein a diagnostic Biomarker for early stage oral Squamous Cell Carcinoma. Pak J Med Sci. 2023;39:513–7. 10.12669/pjms.39.2.6488. 36950446 10.12669/pjms.39.2.6488 PMC10025728

[19] Alharithy RN. Polymorphisms in RETN gene and susceptibility to colon cancer in Saudi patients. Ann Saudi Med. 2014;34:334–9. 10.5144/0256-4947.2014.334. 25811207 10.5144/0256-4947.2014.334 PMC6152568

[20] Vallega KA, Liu N, Myers JS, Yu K, Sang QX. Elevated resistin gene expression in African American estrogen and progesterone receptor negative breast cancer. PLoS One. 2016;11:e0157741 10.1371/journal.pone.0157741. 27314854 10.1371/journal.pone.0157741 PMC4912107

[21] Hu WW, Tang CH, Sun Y, Lu TT, Jiang P, Wu YM, et al. Correlation between resistin gene polymorphism and clinical aspects of lung cancer. Medicine (Baltimore). 2017;96:e9485 10.1097/MD.0000000000009485. 29384942 10.1097/MD.0000000000009485 PMC6392976

[22] Arif K, Shaikh F, Khan R, Ahmed Baig F, Mirza T. The pertinence of resistin gene single nucleotide polymorphism G > A and its expression in oral cancer. Gene. 2025;935:149062 10.1016/j.gene.2024.149062. 39481769 10.1016/j.gene.2024.149062

[23] Yang WH, Wang SJ, Chang YS, Su CM, Yang SF, Tang CH. Association of resistin gene polymorphisms with oral squamous cell carcinoma progression and development. Biomed Res Int. 2018;2018:9531315. 10.1155/2018/9531315. 30406149 10.1155/2018/9531315 PMC6204179

[24] Lu Z, Liu J, Wan Q, Wu Y, Wu W, Chen Y. Chemerin promotes invasion of oral squamous cell carcinoma by stimulating IL-6 and TNF-α production via STAT3 activation. Mol Biol Rep. 2024;51:436 10.1007/s11033-024-09359-y. 38520551 10.1007/s11033-024-09359-y

[25] Ghallab NA, Shaker OG. Serum and salivary levels of chemerin and MMP-9 in oral squamous cell carcinoma and oral premalignant lesions. Clin Oral Investig. 2017;21:937–47. 10.1007/s00784-016-1846-8. 27161218 10.1007/s00784-016-1846-8

[26] Guo XH, Wang JY, Gao Y, Gao M, Yu GY, Xiang RL, et al. Decreased adiponectin level is associated with aggressive phenotype of tongue squamous cell carcinoma. Cancer Sci. 2013;104:206–13. 10.1111/cas.12077. 23181352 10.1111/cas.12077 PMC7657126

[27] Guo XH, Wu MY, Zhao G, Wu FH, Xu YD, Yin MZ, et al. The locoregional adiponectin and its synergistic antitumour effect with HIF-1α blockade in TSCC. Oral Dis. 2023;29:515–27. 10.1111/odi.13948. 34174132 10.1111/odi.13948

[28] Gröschl M, Topf HG, Kratzsch J, Dötsch J, Rascher W, Rauh M. Salivary leptin induces increased expression of growth factors in oral keratinocytes. J Mol Endocrinol. 2005;34:353–66. 10.1677/jme.1.01658. 15821102 10.1677/jme.1.01658

[29] Tsuchiya T, Shimizu H, Horie T, Mori M. Expression of leptin receptor in lung: leptin as a growth factor. Eur J Pharm. 1999;365:273–9. 10.1016/s0014-2999(98)00884-x. 10.1016/s0014-2999(98)00884-x 9988112

[30] Laud K, Gourdou I, Pessemesse L, Peyrat JP, Djiane J. Identification of leptin receptors in human breast cancer: functional activity in the T47-D breast cancer cell line. Mol Cell Endocrinol. 2002;188:219–26. 10.1016/s0303-7207(01)00678-5. 11911959 10.1016/s0303-7207(01)00678-5

[31] Sobrinho Santos EM, Guimarães TA, Santos HO, Cangussu LMB, de Jesus SF, Fraga CAC, et al. Leptin acts on neoplastic behavior and expression levels of genes related to hypoxia, angiogenesis, and invasiveness in oral squamous cell carcinoma. Tumour Biol. 2017;39:1010428317699130 10.1177/1010428317699130. 28459203 10.1177/1010428317699130

[32] Samuel-Mendelsohn S, Inbar M, Weiss-Messer E, Niv-Spector L, Gertler A, Barkey RJ. Leptin signaling and apoptotic effects in human prostate cancer cell lines. Prostate. 2011;71:929–45. 10.1002/pros.21309. 21541970 10.1002/pros.21309

[33] Giordano C, Vizza D, Panza S, Barone I, Bonofiglio D, Lanzino M, et al. Leptin increases HER2 protein levels through a STAT3-mediated up-regulation of Hsp90 in breast cancer cells. Mol Oncol. 2013;7:379–91. 10.1016/j.molonc.2012.11.002.8. 23228483 10.1016/j.molonc.2012.11.002 PMC5528468

[34] Yapijakis C, Kechagiadakis M, Nkenke E, Serefoglou Z, Avgoustidis D, Vylliotis A, et al. Association of leptin -2548G/A and leptin receptor Q223R polymorphisms with increased risk for oral cancer. J Cancer Res Clin Oncol. 2009;135:603–12. 10.1007/s00432-008-0494-z. 18855010 10.1007/s00432-008-0494-z PMC12160154

[35] Hussain SR, Naqvi H, Gupta S, Mahdi AA, Kumari P, Waseem M, et al. A study on oncogenic role of leptin and leptin receptor in oral squamous cell. Tumour Biol. 2015;36:6515–23. 10.1007/s13277-015-3342-1. 25809704 10.1007/s13277-015-3342-1

[36] Hung WC, Tsai CM, Lin CW, Chuang CY, Yang SF, et al. Leptin -2548 G/A polymorphisms are associated to clinical progression of oral cancer and sensitive to oral tumourization in nonsmoking population. J Cell Biochem. 2019;120:15145–56. 10.1002/jcb.28776. 31021458 10.1002/jcb.28776

[37] Kaur J, Jacobs R. Salivary and serum leptin levels in patients with squamous cell carcinoma of the buccal mucosa. Clin Oral Investig. 2016;20:39–42. 10.1007/s00784-015-1472-x. 25875426 10.1007/s00784-015-1472-x

[38] Gharote HP, Mody RN. Estimation of serum leptin in oral squamous cell carcinoma. J Oral Pathol Med. 2010;39:69–73. 10.1111/j.1600-0714.2009.00808.x. 19817969 10.1111/j.1600-0714.2009.00808.x

